# A Moving Ship Detection and Tracking Method Based on Optical Remote Sensing Images from the Geostationary Satellite

**DOI:** 10.3390/s21227547

**Published:** 2021-11-13

**Authors:** Wei Yu, Hongjian You, Peng Lv, Yuxin Hu, Bing Han

**Affiliations:** 1 Aerospace Information Research Institute, Chinese Academy of Sciences, Beijing 100094, China; yuwei@mail.ie.ac.cn (W.Y.); hjyou@mail.ie.ac.cn (H.Y.); lvpeng@aircas.ac.cn (P.L.); han_bing@mail.ie.ac.cn (B.H.); 2 School of Electronic, Electrical and Communication Engineering, University of Chinese Academy of Sciences, Beijing 100049, China; 3 Key Laboratory of Technology in Geo-Spatial Information Processing and Application Systems, Chinese Academy of Sciences, Beijing 100190, China

**Keywords:** geostationary orbit satellites, GF-4 satellites, ship detection, ship tracking, visual saliency, data association

## Abstract

Geostationary optical remote sensing satellites, such as the GF-4, have a high temporal resolution and wide coverage, which enables the continuous tracking and observation of ship targets over a large range. However, the ship targets in the images are usually small and dim and the images are easily affected by clouds, islands and other factors, which make it difficult to detect the ship targets. This paper proposes a new method for detecting ships moving on the sea surface using GF-4 satellite images. First, the adaptive nonlinear gray stretch (ANGS) method was used to enhance the image and highlight small and dim ship targets. Second, a multi-scale dual-neighbor difference contrast measure (MDDCM) method was designed to enable detection of the position of the candidate ship target. The shape characteristics of each candidate area were analyzed to remove false ship targets. Finally, the joint probability data association (JPDA) method was used for multi-frame data association and tracking. Our results suggest that the proposed method can effectively detect and track moving ship targets in GF-4 satellite optical remote sensing images, with better detection performance than other classical methods.

## 1. Introduction

The surveillance of sea-surface ships is highly significant for the economic development of sea areas, marine environmental protection, marine ship management and fishery safety supervision [1]. With the rapid development of aerospace, sensor and computer technologies, satellite remote sensing technology has also developed rapidly and has become an important means of monitoring maritime ships [2]. For many years, synthetic aperture radar (SAR) images have been used to detect and track ships, since these images are not affected by weather or time. Compared with SAR satellite images, optical satellite remote sensing images better reflect the shape of ships, which makes them easier to recognize and interpret manually. In this respect, high-resolution optical remote sensing images from low Earth orbit (LEO) satellites have been used in recent years to detect and identify ships on the sea [3,4,5,6,7,8] since they provide rich information on the shape and texture of ship targets. However, LEO satellites only provide limited coverage and have a long revisit period, which means that they cannot enable the real-time and continuous monitoring of ship targets moving on the sea surface. In contrast, geostationary orbit (GEO) satellites can continuously observe a large area and have other significant advantages, such as a wide observation range and a short observation period [9]. They can thus be used for the near real-time monitoring and tracking of maritime ships to obtain dynamic motion information, such as the position, heading, speed and trajectory of moving ships [10]. In 2015, China launched GF-4, a medium-resolution optical remote sensing satellite, in the geostationary orbit and this has multiple observation modes, such as a gaze mode and cruise mode and it can also conduct near real-time observations of ship targets moving on the sea [11,12,13,14].

At present, the mainstream methods used to detect targets in optical remote sensing images include analyzing the gray statistical features and employing deep learning methods and methods based on visual attention mechanisms. In this respect, gray statistical feature methods [3,14,15] are based on identifying the gray value of ships or their wakes, which are significantly higher than that of the sea surface. However, the accuracy of this method is limited by the existence of clouds and islands, which cause false alarms or missed detection rates. Methods based on deep learning [16,17,18] extract the textural and geometric features of a target. However, the GEO optical images lack textural information about targets and the target detection method based on deep learning cannot be applied to medium- and low-resolution remote sensing images. In contrast, the method based on the visual attention mechanism [19,20] can quickly and accurately extract regions of interest from complex scenes and the contrast mechanism, multi-resolution representation, size adaptation and other characteristics of the human vision system (HVS) make it efficient and robust for small target detection.

The gray value of ship targets in optical remote sensing images is generally much lower than that of clouds and islands. However, owing to the long imaging distance coupled with atmospheric attenuation and cloud occlusion, it is difficult to observe the ship itself in an optical remote sensing image from a GEO satellite with a medium resolution and it is not easy to detect the ship directly. A ship forms a wake behind it as it moves and the area of the wake is always larger than the ship itself. In addition, compared with the sea surface and the hull, a ship’s wake often has stronger optical reflection characteristics and these are a main feature used to identify and detect a moving ship. Therefore, detecting and analyzing the wake of a moving ship is an effective method of detecting its movement on the sea surface. Some studies have researched ship target detection and tracking methods based on GEO optical images [15,21,22,23]. However, these methods do not consider the wake of a moving ship. In addition, cloud and island interference causes false alarms or missed detections.

Data association is another core issue limiting accurate ship tracking. Classical data association methods include nearest neighbor (NN) data association methods, various improved methods based on NN, joint probability data association (JPDA) methods [24] and multiple hypothesis tracking (MHT) methods. One study [21] used the MHT method to achieve multi-frame GF-4 optical satellite remote sensing image ship target-associated tracking while suppressing false alarms and another [22] used the DCF-CSR algorithm to track ship targets in the remote sensing image of a geosynchronous orbit optical satellite. However, considering that track crossing does not occur at the same time for ships moving on the sea, calculations using the MHT method is complicated; hence, the JPDA method is more suitable for ship tracking.

In this study, we analyze the wake characteristics of ships moving on the sea and propose a moving ship detection and tracking method based on the optical remote sensing images from GEO satellites, specifically involving GF-4 satellite panchromatic remote-sensing images. First, the ANGS method is used to enhance the optical remote sensing image, with the aim of suppressing bright cloud and sea clutter in the image and highlighting the dim ship wake. Second, based on the visual saliency theory, we propose a multiscale dual-neighbor difference contrast measure (MDDCM) method, which calculates the saliency map of the image and obtains the location of the salient target. The wake shapes of moving ship targets are analyzed and false targets (such as speckle clouds) are removed from the candidate targets via verifying the shape of the ship’s wake. Finally, the JPDA method is used to conduct data association and multi-target tracking in multi-frame images, with the aim of obtaining the speed and track of real moving ship targets and false targets, such as stationary islands, are also removed.

The following sections provide the following information: in Section 2, we analyze the imaging characteristics of a GF-4 image and the wake characteristics of the moving ship in the image; Section 3 describes the methods proposed in this paper in detail, including the image enhancement method, target detection method and data association method employed; Section 4 presents the experimental results and an analysis of the proposed method; the conclusions are provided in Section 5.

## 2. Analysis of Wake Feature in GF-4 Satellite Optical Remote Sensing Images

GF-4 has a high temporal resolution, a wide coverage and multiple imaging bands. The camera parameters of GF-4 are list in Table 1.

GF-4 images have a spatial resolution of 50 m in the visible band; therefore, a ship that is several hundred meters in length and tens of meters in width occupies only a few pixels in the GF-4 image. In addition, some hulls are coated in stealth materials and lack reflective features, which makes it difficult to detect them directly. Therefore, the characteristics of a ship’s wake are analyzed in this study with the aim of effectively detecting the presence of a ship.

**Figure 1:**
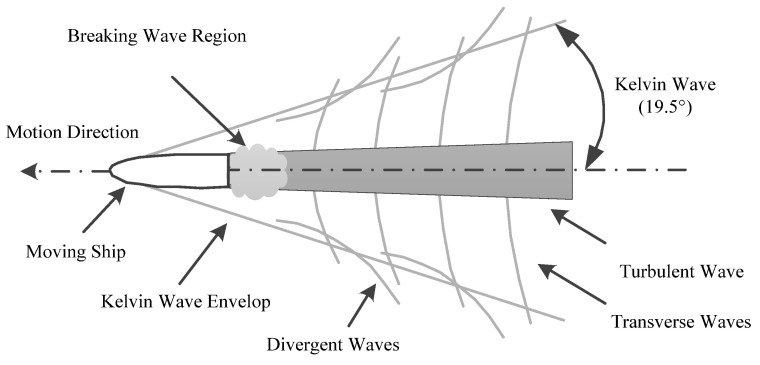
Wake composition of a moving ship.

The wake of a ship is mainly caused by the force between the hull (propeller) and the sea when the ship moves on the sea; this causes subsurface sea water to rise to the surface, which forms the wake. Normally, the width and length of the wake are approximately 1–3 times the width of the ship and 1–20 times the length of the ship, respectively. The wake of a moving ship mainly comprises Kelvin, divergent, transverse, turbulent and breaking waves, as shown in Figure 1. Different wave types of the wake can be identified in different image types. For example, in SAR remote sensing images, Kelvin waves, divergent waves, shear waves and broken waves can be observed; Kelvin waves, divergent waves, shear waves, turbulent waves and broken waves can be observed in high-resolution optical remote sensing images; but only turbulent waves can be observed in GF-4 satellite optical remote sensing images. The observed wake of the turbulent wave appears as a spindle-shaped bright area in the GF-4 image and its brightness and width decrease continuously along the opposite direction of the ship’s motion. The main factors affecting the turbulence wake size in the GF-4 images are the size and sailing speed of the ship.

**Figure 2:**
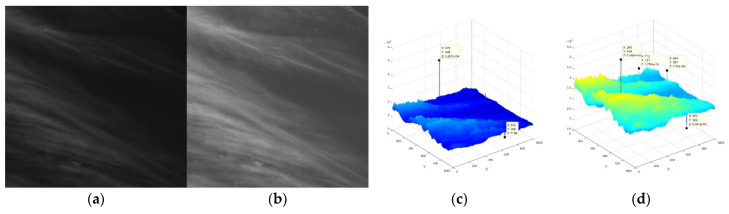
(**a**) 2D view of the original GF-4 satellite image; (**b**) 2D view of the stretched GF-4 satellite image; (**c**) 3D view of the original GF-4 satellite image (**d**) 3D view of the stretched GF-4 satellite image.

Figure 2a shows a two-dimensional (2D) view of the original GF-4 image, Figure 2b shows a 2D view of the stretched GF-4 image and Figure 2c shows a three-dimensional (3D) view of Figure 2a. It is evident that extremely bright flawed pixels appear at (245,448) and dark flawed pixels appear at (672,966). Therefore, it can be concluded that in the GF-4 images, the maximum and minimum gray values are often represented by flawed pixels. In addition, the gray value difference between the clouds and the sea is not obvious and the gray value change is mainly reflected in the floating change in cloud brightness. Furthermore, the ship’s wake is difficult to find. Figure 2b shows a 3D view of the stretched GF-4 image, where the gray difference between the cloud and sea background has been enlarged, the flawed pixels at (245,448) and (672,966) have been suppressed and the ship wakes at (713,131) and (944,291) have been highlighted. This is therefore a better image to use for detecting the ship’s wake.

**Figure 3:**
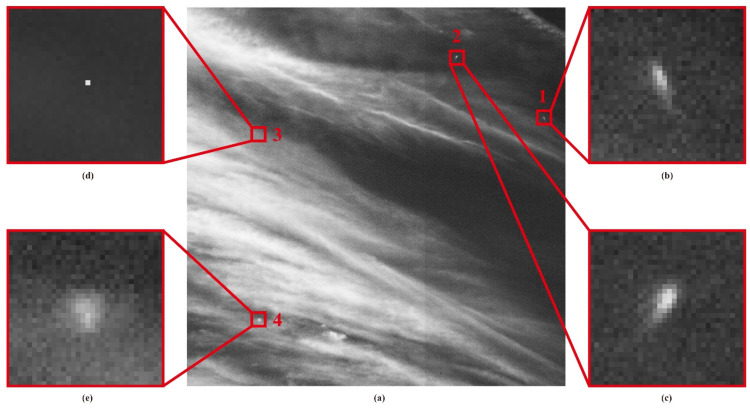
(**a**) Stretched image from GF-4; (**b**) enlarged view of ship’s wake in red box area1; (**c**) enlarged view of ship’s wake in red box area2; (**d**) enlarged view of flawed pixels in red box area3; (**e**) enlarged view of spot-shaped cloud in red box area4.

GF-4 satellite optical remote sensing images can contain cloud clutter, ships’ wakes and flaws, as shown in Figure 3. After analyzing this image, the following were determined: (1) GF-4 satellite optical remote sensing image contain a large number of bright clouds and the gray values of these are higher than that of the ship’s wake; (2) the ship forms an obvious wake as it moves on the sea and the gray values of the wake are higher than the sea background; (3) the wakes of different ships differ in size and brightness in relation to the size and speed of the ship itself; and (4) flawed image pixels from the camera of the GF-4 satellite result from the very harsh electromagnetic radiation environment of the stationary orbit. The remote sensing image contains several bright and black flawed pixels, but there are more pixels representing the ship’s wake than there are flawed pixels; (5) the brightness of the cloud varies, but there are large areas of bright clouds or local bright spot-shaped clouds.

## 3. The Proposed Methods

**Figure 4:**
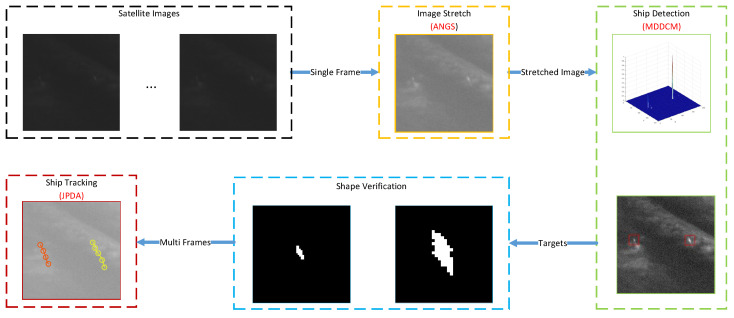
Proposed detection and tracking framework.

The above analysis shows that identifying ship targets in GF-4 satellite optical remote sensing images is problematic due to clouds and flaws. However, the brightness of the ship’s wake is lower than that of clouds but higher than that of sea background and image stretching can enhance the ship’s wake within an image. Although the ship’s wake in the image is a turbulent wake with a certain shape, it remains weak and only occupies a small number of pixels within the image. However, as the GF-4 satellite is a video-like satellite that can continuously scan the observation area and form an image sequence, the multi-frame association method can be used to identify a moving ship and remove false targets. The target detection and tracking framework of sea-surface moving ships proposed in this study are shown in Figure 4.

The method proposed in this paper includes four stages: image enhancement, ship detection, shape verification and multi-frame association and are outlined as follows:

(1) Image enhancement stage: ANGS is used to enhance each frame of the GF-4 image sequence to improve the image contrast and highlight the ship’s wake in the image.
(2) Target detection stage: the focus of this study is to detect targets based on visual saliency. In this respect, the MDDCM method is used to calculate the saliency map and the image is then segmented according to the dynamic threshold value to obtain the location of the candidate ship target.
(3) Shape verification stage: based on the detection results of MDDCM, the region in which the target is located is binarized to obtain the shape of the ship’s wake and remove false targets that do not have ship wake characteristics.
(4) Multi-frame tracking stage: the JPDA method is used for data association to confirm the real moving ship target from the candidate targets in multi-frame images. In addition, the ship’s speed and its track are obtained and false targets, such as stationary islands, are removed.

### 3.1. GF-4 Satellite Optical Remote Sensing Image Enhancement

The analysis above showed that the pixels with the greatest brightness in the GF-4 satellite optical remote sensing image are often flawed and the brightness of cloud is generally higher than that of the ship’s wake. Therefore, to effectively detect ships on the sea surface, it is necessary to enhance the GF-4 image to heighten the contrast between the ship’s wake and the sea background. Image enhancement methods are generally divided into spatial domain enhancement and frequency-domain enhancement methods. However, owing to the uncertain size and shape of a ship’s wake, the frequency-domain enhancement method is not suitable for image enhancement in this context. Therefore, spatial domain image enhancement methods are preferred and these include histogram equalization, Laplace transform, log transform and gamma transform. In this paper, an adaptive nonlinear gray stretch (ANGS) method is used to suppress high-brightness clouds and to simultaneously enhance the brightness of the ship’s wake. The formula for the ANGS is as follows:

(1) $$F\left(x,y\right)=\frac{1}{1+{\left(\frac{m_{gray}}{G\left(x,y\right)+eps}\right)}^{E}}$$

where *G* (*x*, *y*) is the original remote sensing image of GF-4, $m_{gray}$ is the mean value of the original image *G* (*x*, *y*), *E* is the stretch factor that controls the slope of the stretch curve and *eps* is a very small value that prevents the formula from being meaningless when *G* (*x*, *y*) = 0. Furthermore, *E* is an empirical value and different stretching factors of *E* have different stretching effects on the image: the larger the value of *E*, the greater the gray contrast near the mean value $m_{gray}$ and the gray value compression of the high and low gray levels is also stronger.

### 3.2. MDDCM Method for Ship Wake Detection

As previously mentioned, the analysis conducted in Section 2 showed that the gray value of the ship’s wake in the GF-4 image was lower than that of the clouds and the land. A ship’s wake generally occupies only a dozen (or sometimes a few) pixels in a GF-4 image and, thus, it is a small and dim target. In recent years, algorithms based on the human visual system (HSV) have demonstrated a good performance for detecting dim and small targets. HVS algorithms, including the local contrast measure (LCM) method [25] and multiscale patch-based contrast measure (MPCM) method [26], generally use the local gray difference between the target and the surrounding background to calculate the contrast and extract the target position. Based on the HVS theory, we propose a multiscale dual-neighbor difference contrast measure (MDDCM) method for detecting a ship’s wake in GF-4 images.

#### 3.2.1. DDCM Window Structure

**Figure 5:**
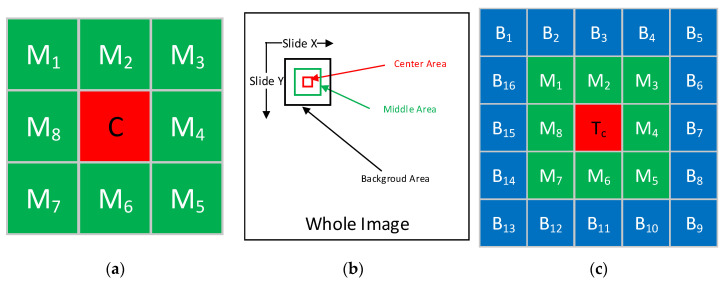
(**a**) Structure of the LCM; (**b**) Slide whole image using DDCM window; (**c**) Structure of DDCM.

The traditional LCM window is shown in Figure 5a. The window includes nine sub-blocks: a central block and the adjacent eight sub-blocks. The traditional LCM algorithm measures the visual salience of the central block by calculating the contrast between the central block and the surrounding eight sub-blocks.

In contrast to the traditional LCM window, we designed a dual-neighborhood window that includes central, middle and background regions. By sliding the dual-neighborhood window onto the entire image, the local contrast of the image can be calculated and a saliency map is then generated. The sliding process is illustrated in Figure 5b. The structure of the dual-neighborhood window is shown in Figure 5c, which contains a central region R_C_, middle region, R_M_ and background region, R_B_. R_C_ contains 1 sub-block, T_c_, R_M_ contains eight middle sub-blocks, M1–M8 and R_B_ contains 16 background sub-blocks, B1–B16.

#### 3.2.2. DDCM Local Contrast Calculation

**Figure 6:**
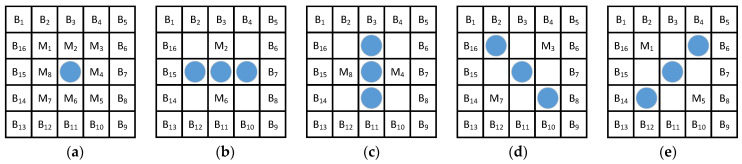
(**a**) Distribution of gaussian spot-like wake in DDCM window; (**b**) Distribution of strip-like with 0° angle in DDCM window; (**c**) Distribution of strip-like with 90° angle in DDCM window; (**d**) Distribution of gaussian strip-like with 135° angle in DDCM window; (**e**) Distribution of gaussian strip-like with 45° angle in DDCM window.

Our previous analysis showed that a ship’s wake appears as a spindle-shaped bright area in a GF-4 image. When the wake is small, it appears as a Gaussian spot and when the wake is large, it appears as a long strip. The distribution of the Gaussian spot-like ship’s wake on the DDCM window is shown in Figure 6a. As the course of the hull is arbitrary, we use four angles, as shown in Figure 6b–e, to roughly describe the distribution of the strip-like ship’s wake in the DDCM window.

Based on the DDCM window structure, the difference between the three regions is used to measure the local contrast. The contrast between the central sub-block, T_c_ and the middle region of each sub-block, $M_{i}$, is represented by $d(T,M_{i})$ and the expression of $d(T,M_{i})$ is

(2) $$d(T,M_{i})=\{\begin{array}{ll} mean_{T}-mean_{M_{i}} & \;\mathrm{if}\;mean_{T}-mean_{M_{i}}>0\\ 0 & \;\mathrm{else}\; \end{array},i=1,2,3...8$$

where $mean_{T}$ represents the gray mean of the central sub-block, T_c_; $mean_{M_{i}}$ represents the gray mean of the middle sub-block, $M_{i}$; and *i* = 1, 2, 3...... 8.

The contrast between the central sub-block, T_c_ and the background region is represented by $D_{B}$ and the expression of $D_{B}$ is

(3) $$D_{B}=\{\begin{array}{ll} mean_{T}-\max(mean_{B_{k}}) & \;\mathrm{if}\;mean_{T}-\max(mean_{B_{k}})>0\\ 0 & \;\mathrm{else}\; \end{array},k=1,2,3...16$$

where $mean_{T}$ represents the gray mean of the central sub-block, T_c_; $mean_{B_{k}}$ represents the gray mean of the background sub-block $B_{k}$, where *k* = 1, 2, 3... 16; and $\max(mean_{B_{k}})$ represents the maximum value of $mean_{B_{k}}$ in 16 background subblocks. The gray value of the central region where the ship wake is located is generally higher than that of the outer background region; therefore, by identifying $D_{B}$, cloud clutter can be effectively suppressed and the ship’s wake can be highlighted.

A ship’s wake is generally distributed symmetrically along the central axis. When the central sub-block of the DDCM window passes through the center of the ship’s wake, a part of the ship’s wake may be distributed in the middle region. Therefore, the contrast between the central region, R_C_ and the middle region, R_M_, can be expressed as $D_{m}$, where the expression of $D_{m}$ is as follows,

(4) $$D_{M}=\{\begin{array}{l} d(T,M_{3})\times d(T,M_{7})\;\;\;\;\mathrm{if}\;\;\max(mean_{M_{i}})=mean_{M_{1}}\;\mathrm{or}\;\;\max(mean_{M_{i}})=mean_{M_{5}}\\ d(T,M_{4})\times d(T,M_{8})\;\;\mathrm{else}\;\mathrm{if}\;\;\max(mean_{M_{i}})=mean_{M_{2}}\;\mathrm{or}\;\;\max(mean_{M_{i}})=mean_{M_{4}}\\ d(T,M_{5})\times d(T,M_{1})\;\;\mathrm{else}\;\mathrm{if}\;\;\max(mean_{M_{i}})=mean_{M_{3}}\;\mathrm{or}\;\;\max(mean_{M_{i}})=mean_{M_{7}}\\ d(T,M_{6})\times d(T,M_{2})\;\;\mathrm{else}\;\mathrm{if}\;\;\max(mean_{M_{i}})=mean_{M_{4}}\;\mathrm{or}\;\;\max(mean_{M_{i}})=mean_{M_{8}} \end{array}$$

where $\max(mean_{M_{i}})$ represents the maximum value of $mean_{M_{i}}$ for the middle region of eight sub-blocks and the sub-block corresponding to $\max(mean_{M_{i}})$ may be the sub-block where the ship’s wake symmetry axis is located. Therefore, we use the sub-block perpendicular to the sub-block of $\max(mean_{M_{i}})$ to calculate the contrast between the central region, R_C_ and the middle region, R_M_.

If the size of the central region, R_C_, in the DDCM window is *k* × *k*, the visual saliency of the *k*-size DDCM can be expressed by ${\mathrm{DDCM}}_{k}$ as follows:

(5) $${\mathrm{DDCM}}_{k}=D_{m}\times D_{B}$$

where $D_{m}$ is the contrast between the central and middle regions and $D_{B}$ is the contrast between the central region and the background region. By setting the value of the DDCM window size *k* and using the DDCM window to slide through the entire GF-4 image, a single-scale DDCM saliency map is obtained.

#### 3.2.3. MDDCM Local Contrast Calculation

The size of a ship’s wake varies in accordance with factors such as the hull size, sailing speed and the spatial resolution of the image that it is caught within. Therefore, a single-scale DDCM window is not suitable for detecting ships’ wakes of all sizes. We thus use the multiscale dual-neighbor difference contrast measure (MDDCM) method to detect the wake of a ship.

The DDCM saliency map of size k can be represented as ${\mathrm{DDCM}}_{k}$; the saliency of a pixel P(i,j) in the image can be represented as ${\mathrm{DDCM}}_{k}(i,j)$; and the expression of the saliency ${\mathrm{MDDCM}}_{k}(i,j)$ of pixel P(i,j) in the MDDCM algorithm is as follows:

(6) $${\mathrm{MDDCM}}_{k}(i,j){=\max(\mathrm{DDCM}}_{k}(i,j)),\;k=2,3,4,5...K$$

where *k* is the size of the DDCM and the minimum and maximum values are 2 and K, respectively. Therefore, the MDDCM with the largest scale of K can be expressed as

(7) $${\mathrm{MDDCM}=\max(\mathrm{DDCM}}_{k}),\;k=2,3,4,5...K$$

#### 3.2.4. Adaptive Extraction of Ship’s Position Based on MDDCM

Following the MDDCM calculation, an MDDCM saliency map of the image can be obtained. The contrast of the area of the ship’s wake is enhanced, while bright flaws, random noise, cloud clutter and sea background clutter are effectively suppressed and the signal-to-noise ratio (SNR) is significantly improved. To extract the target, we normalize the saliency map to distribute it between 0 and 1 and then use an adaptive threshold to segment the saliency map to obtain the location of the target. The expression of threshold τ used for saliency map segmentation in this study is

(8) $$\tau =\mu +\lambda_{th}\sigma ,$$

where *μ* is the mean value of the MDDCM saliency map, σ is the standard deviation of the MDDCM saliency map and $\lambda_{th}$ is the segmentation factor, which typically ranges from 20 to 50. Using the threshold τ to segment the saliency map, the position of the target, (the candidate ship) is extracted.

### 3.3. Shape-Based False Alarm Removal

The detection result of the MDDCM saliency map provides the position of the candidate ship in the image, but they do not show the size and shape of the candidate ship. In fact, the extraction result in the MDDCM saliency map is usually the central position of the ship’s wake. Therefore, after obtaining the location of the ship using MDDCM, it is necessary to segment the target candidate region to contour information about the candidate ship. Shape verification can then be used to remove false targets, such as spot-shaped clouds.

Based on the location of the candidate target extracted by MDDCM, we extracted 64 × 64-pixel regions of interest (ROI) and then used the Otsu method to perform binary segmentation of the ROI to separate the target from the sea surface background. The formula is as follows:

(9) $$F(x,y)=\{\begin{array}{l} 1,F(x,y)\geq Th_{\mathrm{otsu}}\\ 0,F(x,y)<Th_{\mathrm{otsu}} \end{array}$$

where $Th_{\mathrm{otsu}}$ is the segmentation threshold of Otsu’s method. For the segmented image, the connected region centered on the detection result of the MDDCM saliency map is the region of the ship. However, the detection results of the MDDCM saliency map often contain false alarms, but through binary segmentation of the target area, the target contour can be obtained and the target’s shape characteristics can then be verified.

The spatial resolution of the GF-4 satellite optical remote sensing image is 50 m and the width of a ship is generally within 50 m (not exceed 100 m). According to the above analysis, the width of the ship’s wake is approximately one to three times that of the hull and the wake’s length is one to twenty times the length of the hull. Therefore, the width of the ship’s wake in the GF-4 image occupies only a few pixels but its length can be dozens of pixels. Generally, the larger the wake’s width, the larger the size of the ship or the faster its speed; this means that the turbulent wake formed behind the ship will be longer and there is thus a positive correlation between the length and width of the ship’s wake. Based on this feature, we can verify the shape of targets and remove false alarm targets that do not meet the shape characteristics of the wake. If the length of the smallest enclosing ellipse of the target area is $Length_{T}$ and the width is $Width_{T}$, the length-to-width ratio is represented by $R_{real}$. Therefore, the width $Width_{T}$ and length-to-width ratio $R_{real}$ of the wake of the ship should have a certain range. We use the following formula to verify the shape of the ship’s wake as follows:

(10) $$Width_{\min}<Width_{T}<Width_{\max}$$

(11) $$R_{th}=\frac{Width_{T}+1}{2}$$

(12) $$\{\begin{array}{c} R_{real}=\frac{Length_{T}}{Width_{T}}\\ R_{real}>R_{th} \end{array},$$

where $Width_{\min}$ and $Width_{\max}$ are the minimum and maximum value ranges of the wake widths, respectively. Limiting the value of $Width_{T}$ can remove small false targets, such as flaws and large false targets, such as clouds and islands. $R_{th}$ is the adaptive threshold value associated with $Width_{T}$. False targets that do not meet wake characteristics can be removed using $R_{th}$.

### 3.4. Ship Tracking Based on JDPA

GEO satellites can detect and track marine moving ships to obtain their moving tracks and estimate their movement trends. In addition, data association can be used to remove false alarm targets, such as stationary islands, irregular clouds and random noise.

As previously mentioned, JPDA is a classic data association method [24]. The main idea is to comprehensively consider all the targets and measurements, according to the association between all measurements and all targets.

In the JPDA method, the predicted state equation of the target is defined as

(13) $${\hat{X}}^{t}(k|k-1)=F^{t}(k-1){\hat{X}}^{t}(k-1|k-1),$$

where ${\hat{X}}^{t}(k|k-1)$ represents the state vector of t at time k predicted by time k−1 and $F^{t}(k-1)$ is the state transition matrix of t at time k−1.

The prediction vector of measurement target is defined as

(14) $${\hat{Z}}^{t}(k|k-1)=H(k){\hat{X}}^{t}(k|k-1),$$

where H(k) represents the measurement target matrix.

According to the Kalman filter formula, the equation for calculating the status update is as follows,

(15) $${\hat{X}}^{t}(k|k)={\hat{X}}^{t}(k|k-1)+K^{t}(k)V^{t}(k),$$

where $K^{t}(k)$ represents the Kalman gain matrix of t at time k and $V^{t}(k)$ represents the combined information of t at time k.

The associated area is defined as

(16) $$A(k)={\left[z\left(k\right)-\hat{z}\left(k|k-1\right)\right]}^{T}S^{-1}\left(k\right)\left[z\left(k\right)-\hat{z}\left(k|k-1\right)\right]\leq \gamma ,$$

where $S^{-1}\left(k\right)$ represents the innovation covariance matrix at time k and γ is a fixed threshold that can be obtained from the $\chi^{2}$ distribution table.

As an extension and optimization of the probability data association (PDA) method, the JPDA method mainly introduces the concept of joint events within the association cycle,

(17) $$\theta_{i}\left(k\right)=\overset{Num_{k}}{\underset{j=1}{\cap }}\theta_{jt}^{i}\left(k\right),$$

where $\theta_{i}\left(k\right)$ is the joint-associated event, k is the time, $\theta_{i}\left(k\right)$ is the ith joint event at time k,$Num_{k}$ represents the total number of measured targets at time k and $\theta_{jt}^{i}\left(k\right)$ represents the event when the measured target, j, is related to track t at time k in the joint event of i. When t=0, there are no related events for the measured target, j and this indicates a false alarm.

**Figure 7:**
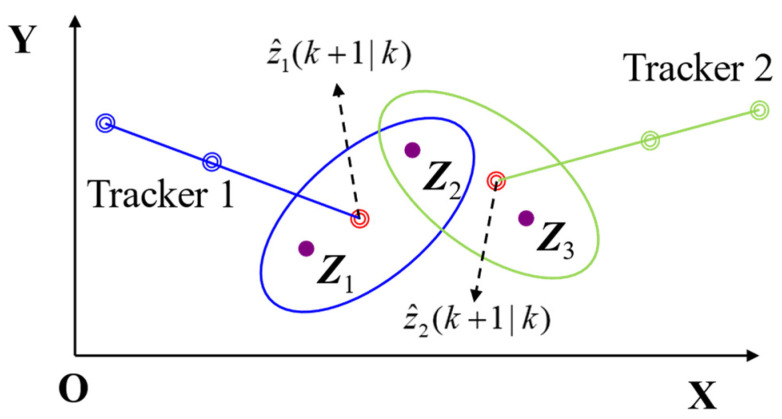
Implementation process of joint probability data association.

Figure 7 illustrates the implementation process of JPDA, which first establishes a confirmation matrix and lists all possible events based on the criterion that the target provides a maximum of one piece of measurement information in a period; the measurement has only one source. Finally, all events are connected via probability to obtain the final status update value for each target.

The JPDA algorithm uses the measurement in the current scan period within the tracking threshold to calculate the correlation probability between the measurement and the corresponding track. The calculation determines the set of all possible “measurement-track” combinations and the probability of the associated set.

We use the target location extracted using the MDDCM method as the data source for data association (the shape has been verified). The JDPA is then used to associate the target data in the multi-frame GF-4 image with the aim of obtaining tracking information about the ship target and calculating its average speed. As the candidate targets in the GF-4 satellite image sequences often contain false targets, we use constraints (such as the minimum associated frames, maximum target speed, minimum target speed and minimum moving distance) to further remove false targets and filter the displacement of the stationary targets caused by camera shake.

## 4. Experiment and Analysis

### 4.1. Experimental Data

**Figure 8:**
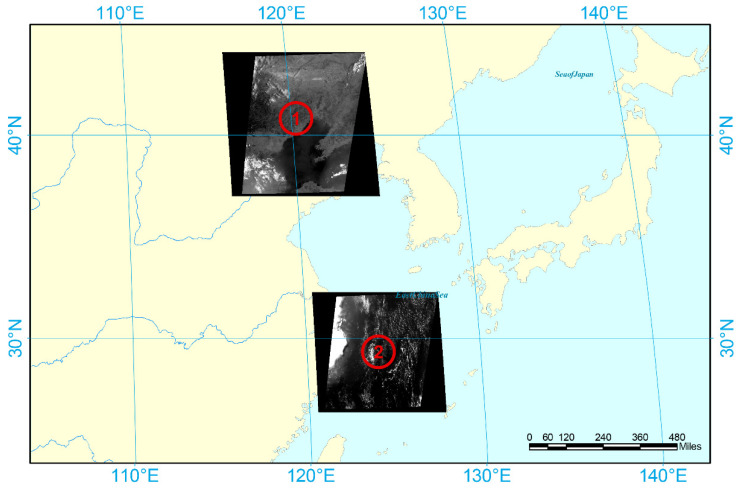
Area where the remote sensing image is located.

Remote sensing images of the two regions were used for experimental data sources and the region where the two remote sensing images are located is shown in Figure 8. The imaging time and latitude and longitude information of remote sensing images are listed in Table 2

To better observe the ship wake, analyze the experimental results and verify the performance of the method, four groups of image slices with different sizes and scenes were extracted from the original remote sensing images as experimental data. A description of the experimental data is presented in Table 3.

**Figure 9:**
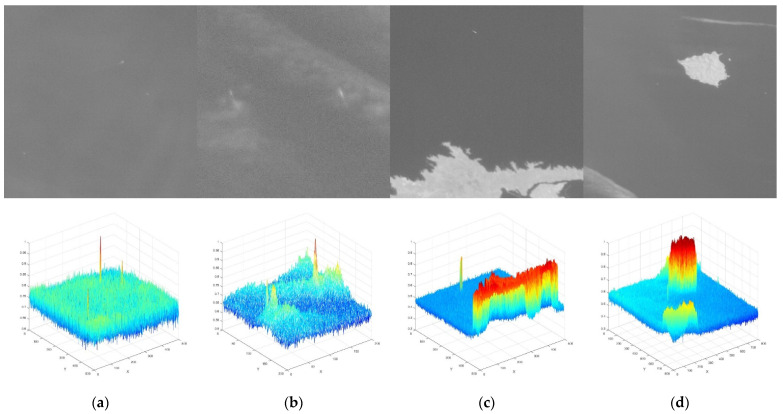
(**a**) 2D and 3D views of stretched images without clouds and island; (**b**) 2D and 3D views of stretched images with clouds; (**c**) 2D and 3D views of stretched images with island; (**d**) 2D and 3D views of stretched images with island and clouds.

In accordance with our detection and tracking framework, ANGS was used to stretch single-frame images to improve the image contrast and highlight the ship targets prior to detecting ship targets in the single-frame remote sensing images. The results of our stretching experiments conducted on several groups of GF-4 satellite panchromatic optical remote sensing images, the stretching effect was superior when the value of *E* was set at between 6 and 8. We therefore set the parameter *E* to 6 in the experiment. The stretched images of the four groups of experimental data are illustrated in Figure 9.

### 4.2. Detection and Comparison

To verify the performance of this method, we conducted comparative experiments between using the MDDCM method and other visual saliency methods, including the multi-scale patch-based contrast measure (MPCM) method [26], the local peak signal-to-noise ratio (PSNR) method [21] and the spectral residual (SR) method based on the frequency domain [27].

#### 4.2.1. Experimental Parameters

The range of the MDDCM window size depends on the image resolution and the target size. Our previous analysis showed that the width of the wake in a GF-4 image was few pixels, while the length could reach dozens. Therefore, the size *k* of the MDDCM window was set in the range of 2–4 for the multi-scale saliency map calculation.

Similarly, the size of the MPCM window depends on the size of the target. In addition, MPCM method supports multiple scales; hence, we set the K value of MPCM to 3 and 5.

The convolution kernel size of SR method is related to the target size and can only be set to one value. Through experiments, we found that when *n* is 3, the detection result is better.

In article [21] for PSNR method, the author explicitly proposed that the value of *K_out_* is 20 and the value of *K_in_* is 10. In addition, fixed threshold *th* is adopted for segmentation and the authors suggested *th* to be set at 3–5, therefore, here, *th* was 4.

The segmentation threshold τ was used to segment the saliency map. The segmentation factor $\lambda_{th}$ is an empirical value, usually 20–50. Except for the PSNR method, the value of $\lambda_{th}$ was uniformly set to 20 to compare and verify the performance of each method.

The experimental parameter settings of each method are listed in Table 4.

#### 4.2.2. Comparison

**Figure 10:**
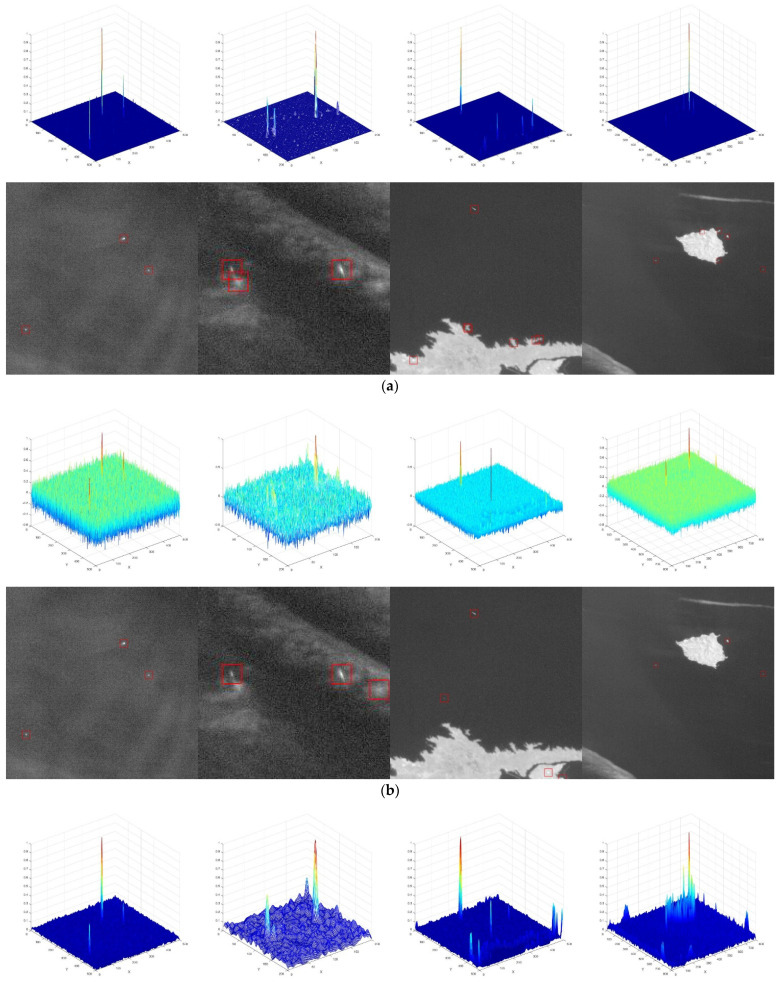

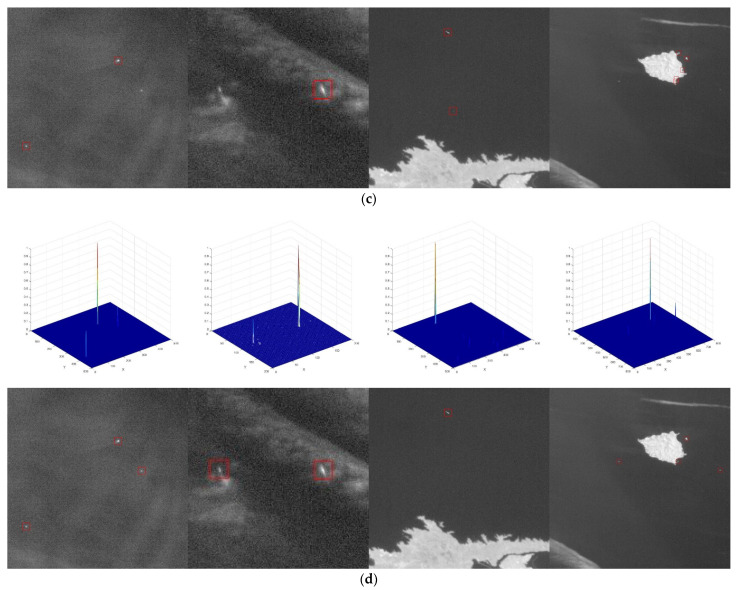
(**a**) 3D view of saliency map and ship detection result using MPCM; (**b**) 3D view of saliency map and ship detection result using PSNR; (**c**) 3D view of saliency map and ship detection result using SR; (**d**) 3D view of saliency map and ship detection result using MDDCM.

The comparative experimental ship detection results using the different methods with the four groups of experimental data introduced above are shown in Figure 10.

Figure 10a shows that the MPCM method has a good background suppression ability. The detection performance of small targets is very good and all small targets in the four scenes can be detected; but in the presence of clouds or islands, there are several false alarms, while the anti-interference ability becomes insufficient. In Figure 10b, the detection performance of the PSNR method is also good, but the background suppression ability is relatively poor and there are many false alarms in the data of group 2 and group 3.

Figure 10c shows that SR method misses targets in groups 1, 2 and 4 data and the detection performance of small targets is poor. False alarms appeared in the data of groups 3 and 4.

It can be seen from Figure 10d that our MDDCM method successfully detected all targets with a low false alarm rate and excellent background suppression ability. This greatly improved the SNR and it provided the best detection performance of all the methods used in comparison. Although false alarms occurred in the fourth group of data, these false alarms can be subsequently removed using the follow-up method.

### 4.3. Shape-Based False Alarm Removal

Based on the image resolution analysis and the size and shape of the ship’s wake, we set $Width_{\min}$ and $Width_{\max}$ to 2 and 6, respectively. The aim of shape verification is to further remove false alarms from candidate ship targets and particularly to avoid the interference of spot-shaped clouds in target detection. Table 5 lists the shape verification results for some of the candidate ship targets.

**Figure 11:**
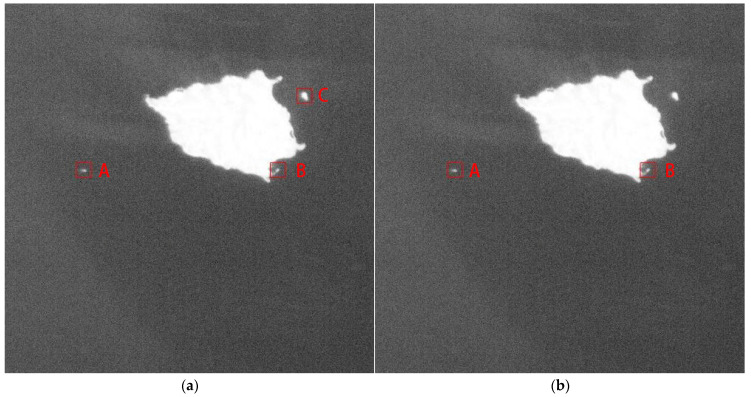
(**a**) Detection result of MDDCM; (**b**) false alarm removal by conducting shape verification. (A, B and C in the figure are three candidate ship targets).

Candidate ship targets detected by the MDDCM algorithm often contain false alarms, such as small islands and spot-shaped clouds and these tend to have high brightness. Figure 11a shows the detection results of the MDDCM algorithm, where target C is an island and target A is a moving ship. The $R_{real}$ of target C does not meet the threshold, $R_{th}$ and it is thus removed from the candidate target. The result is then shown in Figure 11b.

### 4.4. Ship Tracking

After target detection and shape verification within a single frame image in the image sequence, the candidate ship can be obtained in the single frame image. Taking the center pixel of each candidate target as the data source, multi-frame data association of the image sequence can be conducted to obtain the track and speed of the ship. Simultaneously, constraint parameters such as the minimum number of associated frames, maximum speed, minimum speed and minimum moving distance can be used to further remove false targets. The corresponding parameter settings are presented in Table 6.

**Figure 12:**
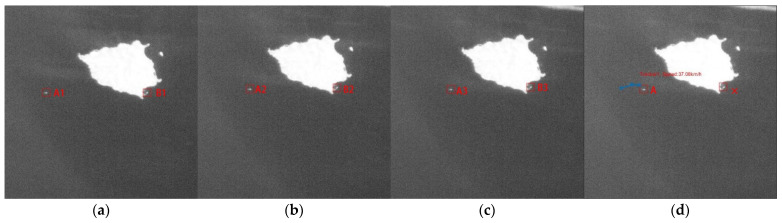
(**a**) Frame 1 with targets A1 and B1 (**b**) frame 2 with targets A2 and B2 (**c**) frame 3 with targets A3 and B3 (**d**) data association result with real target A and false target B.

Figure 12 shows the process involved in associating three frames of data using the JPDA method. The previous method detected two targets, A1 and B1 in Figure 12a, A2 and B2 in Figure 12b and A3 and B3 in Figure 12c. The track and speed of target A were successfully obtained by associating three frames of data. However, the false alarm target B was a fixed island, which was not associated with a stable track, and it was therefore filtered out.

The JPDA algorithm was then used to conduct data association for the candidate targets detected by the five groups of experimental data and the obtained tracking information is shown in Table 7.

### 4.5. Results Evaluation

To verify the effectiveness and performance of the proposed method, we used the recall, precision and F-score to evaluate the method and compare it with other methods. Recall represents the effectiveness of the detection, Precision represents the accuracy of the detection and F-Score is the comprehensive response of recall and precision and the formulas used to calculate them are as follows:

(18) $$Recall=\frac{N_{TP}}{N_{TP}+N_{FN}},$$

(19) $$Precision=\frac{N_{TP}}{N_{TP}+N_{FP}},$$

(20) $$False-Alarm=\frac{N_{FP}}{N_{TP}+N_{FP}},$$

(21) $$F-Score=\frac{2\times Rcall\times Precision}{Rcall+Precision},$$

where $N_{TP}$ and $N_{FN}$ are the number of correctly detected targets and the number of ships that have not been detected, respectively, and $N_{FP}$ is the number of false targets detected. In accordance with the previous experimental data obtained and the experimental parameters, the detection results of the five groups of experimental data were statistically analyzed and the results of a comparison between the proposed MDDCM method and other visual saliency methods are shown in Table 8.

The experimental results show that SR provided the worst performance of all methods and the proposed MDDCM method provided the best performance. The MPCM and MDDCM methods both provided high recall, but the MPCM method identified a greater number of false targets.

Although the MDDCM method provided the highest recall of all methods, which reached 94.8%, its precision was only 70.7% and many false targets were identified. Therefore, the proposed shape verification method was used to further remove false targets that did not have ship’s wake characteristics. The JPDA was then used to conduct a multi-frame data association of candidate targets in a single frame image and false targets could be further removed, if they were not associated with a stable track. These steps show that the detection precision of the MDDCM method can be improved. Table 9 shows the improvements made in the detection performance after shape verification and multi-frame data association.

The experimental results show that after shape verification, some false targets were further filtered and the precision increased from 70.7% to 87.1%. After multi-frame data association, most of the false targets were removed and the false alarm rate was only 1.9%. However, some real targets were filtered out during shape verification and some were discarded because they were not associated with a stable track, which resulted in a slight decrease in the recall rate. Nevertheless, the comprehensive evaluation F-score increased from 81.0% to 95.6%.

## 5. Conclusions

Geostationary optical remote sensing satellites can provide continuous observations of fixed areas. The GF-4 satellite has the advantages of a high temporal resolution and large coverage, which enables continuous observation of ships moving on the sea surface. However, owing to the spatial resolution of the image, as well as interference from high-brightness clouds, islands and sea clutter, ship targets are dim and small in the image, which makes it very difficult to directly detect ships.

We analyzed the wake of a moving ship and proposed a moving ship detection and tracking method based on visual salience. Four groups of GF-4 panchromatic remote sensing images of different scenes and four groups of real GF-4 panchromatic remote sensing images were used in this study. The four groups of data included a cloudless scene, a scene containing dense cloud, a scene containing islands and a complex scene containing both clouds and islands

Through experimental analysis and comparisons with other methods, the proposed method provides a good detection performance, which has a higher recall rate of 93.1% and a lower false alarm rate of 1.9%. Thus, it can be used to effectively detect and track moving ships on the sea surface in GEO optical remote sensing satellite images, even in the presence of dense clouds and islands. Nevertheless, our method has limitations. In the presence of numerous moving spot-shaped clouds, the detection performance will sharply decline, resulting in high false alarm.

Finally, we analyzed the general influencing factors on ship detection in optical remote sensing images, such as clouds and islands and proposed an MDDCM method suitable for small target detection. In low and medium resolution optical satellite remote sensing images, ship targets are usually small and generally affected by bright clouds and islands. Therefore, we will apply the proposed method to other medium-resolution and low-resolution optical satellite remote sensing images for further research.

## Figures and Tables

**Table 1:** The camera parameters of GF-4.

Band		Spectral Range (um)	Spatial Resolution (m)	Swath (km)	Revisit Time (s)	Image Size (pixel)	Digitalizing Bit
Visible and Near Infrared	B1	0.45–0.90	50	400 × 400	20	10,000 × 10,000	16
	B2	0.52~0.60					
	B3	0.63~0.69					
	B4	0.76~0.90					
	B5	0.45~0.52					
Middle Wave Infrared	B6	3.5~4.1	400			1000 × 1000	

**Table 2:** Remote sensing image for experiment.

Region	Band	Imaging Time	Longitude Range	Latitude Range
① Bohai area	B1	2019-03-31 09:00	[116.1223° E, 124.8828° E]	[36.9983° N, 44.1268° E]
② East China Sea area	B1	2020-07-26 15:00	[120.4694° E, 127.7919° E]	[26.3600° N, 32.2755° N]

**Table 3:** Data used in the experiment.

Sequence ID	Spatial Resolution (m)	Temporal Resolution (s)	Image Size (pixel)	Number of Images	Features of the Scene
Sequence1	50	20	500 × 500	8	No Cloud + No Island
Sequence2	50	20	200 × 200	5	With Cloud
Sequence3	50	20	500 × 500	8	With Island
Sequence4	50	20	800 × 800	8	Island + Cloud

**Table 4:** Experimental parameter values of visual saliency methods.

Method	Symbol	Parameter Description	Value
MPCM	*K*	Size of center window	3, 5
	$\lambda_{th}$	Segmentation factor	20
PSNR	*K_in_*	Size of inner window	10
	*K_out_*	Size of outer window	20
	*th*	Fixed segmentation threshold	4
SR	*n*	Size of convolution kernel	3
	$\lambda_{th}$	Segmentation factor	20
MDDCM	*K*	size of center window	2, 3, 4
	$\lambda_{th}$	Segmentation factor	20

**Table 5:** Shape-based verification.

No.	Target	Width	Length	R*_real_*	R*_th_*	Verification
1	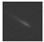	2.6277	11.0888	4.2200	1.8138	True
2	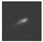	3.1409	8.6349	2.7492	2.0704	True
3	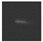	3.2342	10.2844	3.1799	2.1171	True
4	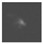	4.4772	7.1736	1.6022	2.7386	False
5	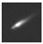	3.6485	12.4416	3.4100	2.3243	True
6	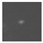	3.0827	4.9899	1.6187	2.0414	False
7	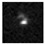	6.2077	10.1860	1.6409	3.6038	False
8	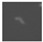	3.8072	8.6802	2.1068	2.4036	False
9	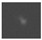	5.0590	7.0992	1.4033	3.0295	False
10	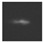	3.0048	13.3384	4.4391	2.0024	True

**Table 6:** Constraint parameters for ship tracking.

Item	Value
Minimum association frames	3
Minimum speed	10 km/h
Maximum speed	80 km/h
Minimum distance	2 km

**Table 7:** Tracking results.

Image Sequence	Total Frames	Track Id	Association Frames	Mean Speed (km/h)
Image Sequence 1	8	Track 1	8	36.12
		Track 2	8	34.77
		Track 3	7	26.46
Image Sequence 2	5	Track 1	5	49.06
		Track 2	4	44.95
Image Sequence 3	8	Track 1	8	58.44
Image Sequence 4	8	Track 1	7	37.08
		Track 2	6	28.62

**Table 8:** Comparison between the performance of MDDCM and those of other methods.

Method	N_TP_ + N_FN_	N_TP_ + N_FP_	N_TP_	N_FN_	N_FP_	Recall (%)	Precision (%)	False-Alarm (%)	F-Score (%)
MPCM	58	124	55	3	69	94.8	44.4	55.6	60.5
PSNR		90	50	8	40	86.2	55.6	44.4	67.6
SR		64	24	26	40	41.4	37.5	62.5	39.4
MDDCM		75	55	3	22	94.8	70.7	29.3	81.0

**Table 9:** Performance improvement by employing shape verification and data association.

Method	Recall (%)	Precision (%)	False-Alarm (%)	F-Score (%)
MDDCM	94.8	70.7	29.3	81.0
After shape verification	93.1	87.1	12.9	90.0
After data association	93.1	98.2	1.9	95.6

## Data Availability

Not applicable.
